# Synthesis and crystal structure of a new alluaudite-like iron phosphate Na_2_CaMnFe(PO_4_)_3_

**DOI:** 10.1107/S2056989016017771

**Published:** 2016-11-15

**Authors:** Semeh Jebli, Abdessalem Badri, Mongi Ben Amara

**Affiliations:** a Unité de Recherche, Matériaux Inorganiques, Faculté des Sciences, Université de Monastir, 5019, Monastir, Tunisia

**Keywords:** XRD, iron phosphate, alluaudite structure, crystal structure

## Abstract

A new iron phosphate, disodium calcium manganese(II) iron(III) tris(phosphate), Na_2_CaMnFe(PO_4_)_3_, has been synthesized as single crystals by the flux technique. This compound crystallizes in the monoclinic space group *C*2/*c*. The structure belongs to the alluaudite structural type and thus, it obeys the *X*(2)*X*(1)*M*(1)*M*(2)_2_(PO_4_)_3_ general formula. Both the *X*(2) and *X*(1) sites are fully occupied by sodium, while *M*(1) is occupied by calcium and *M*(2) exhibits a statistical distribution of iron and manganese.

## Abstract 2

Disodium calcium manganese iron tris­(phosphate), Na_2_CaMnFe(PO_4_)_3_, crystallizes in the monoclinic space group *C*2/*c*. The structure belongs to the alluaudite structural type and thus, it obeys the *X*(2)*X*(1)*M*(1)*M*(2)_2_(PO_4_)_3_ general formula. Both the *X*(2) and *X*(1) sites are fully occupied by sodium, while *M*(1) is occupied by calcium and *M*(2) exhibits a statistical distribution of iron and manganese.

## Chemical context   

A promising line of research in the materials science field is the creation of materials based on inorganic phosphates, which have considerable potential for use in laser engineering, optics and electronics owing to their non-linear optical, electrical and luminescent properties. In recent years, iron monophosphates have assumed great importance for their promising applications in several fields such as catalysis (Moffat, 1978 ▸), corrosion inhibition (Meisel *et al.*, 1983 ▸) and electrochemistry as a positive electrode for lithium ion batteries (Padhi *et al.*, 1997 ▸; Ravet *et al.*,2005 ▸; Trad *et al.*, 2010 ▸). The physical properties of inorganic materials are related to their structure. A large number of iron phosphates belong to the alluaudite structure type (Yakubovich *et al.*, 1977 ▸; Corbin *et al.*, 1986 ▸; Korzenski *et al.*, 1998 ▸; Hatert *et al.*, 2003 ▸; Strutynska *et al.*, 2013 ▸) discovered for the first time from natural minerals by Fisher (1955 ▸). The term alluaudite refers to a large family of natural or synthetic compounds with the general formula proposed by Moore (1971 ▸) of *X*(2)*X*(1)*M*(1)*M*(2)_2_(PO_4_)_3_ with *X* and *M* being cationic sites ranked in descending order of size. The *M* sites are fully occupied while the *X* sites can be empty or partially occupied. In this paper, we report a structural study of a new composition of alluaudite-like iron phosphate Na_2_CaMnFe(PO_4_)_3_. In this compound the *M*(1) and *M*(2) sites are occupied by Ca and (0.5Mn + 0.5Fe), respectively, while the *X*(1) and *X*(2) sites are fully occupied by Na atoms.

In iron phosphates adopting the alluaudite-type structure, the *M*(2) site is often preferentially occupied by iron with oxidation state +III. Consequently, and on basis of the Mössbauer spectroscopy results observed in similar compounds, the presence of Fe^II^ and Mn^III^ in the *M*(2) site was not considered in the Na_2_CaMnFe(PO_4_)_3_ compound. Indeed, in Na_2_Mn_2_Fe(PO_4_)_3_ (Hidouri *et al.*, 2011 ▸), iron and manganese adopt exclusively the oxidation states +III and +II, respectively, whereas in NaMnFe_2_(PO_4_)_3_ (Trad *et al.*, 2010 ▸), Mn^III^ and Fe^II^ were observed in very low amounts, leading to a Mn/Fe ratio close to 1.

## Structural commentary   

**Figure 1:**
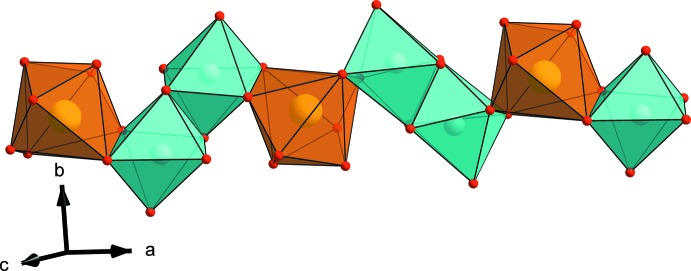
View of a chain showing the distorted octa­hedral sites *M*(1) (orange polyhedra) and *M*(2) (cyan polyhedra).

**Figure 2:**
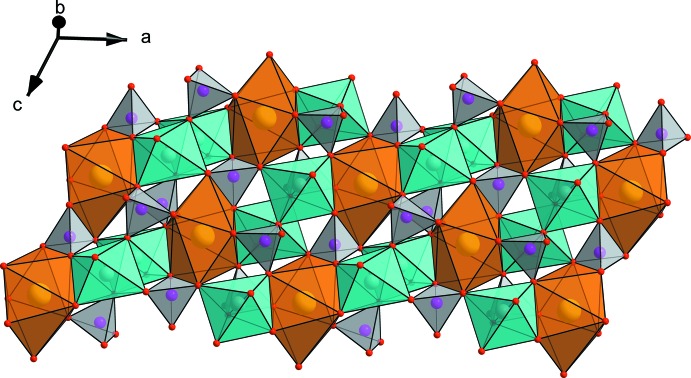
View showing a sheet made of *M*O_6_ octa­hedra and PO_4_ tetra­hedra (light grey).

**Figure 3:**
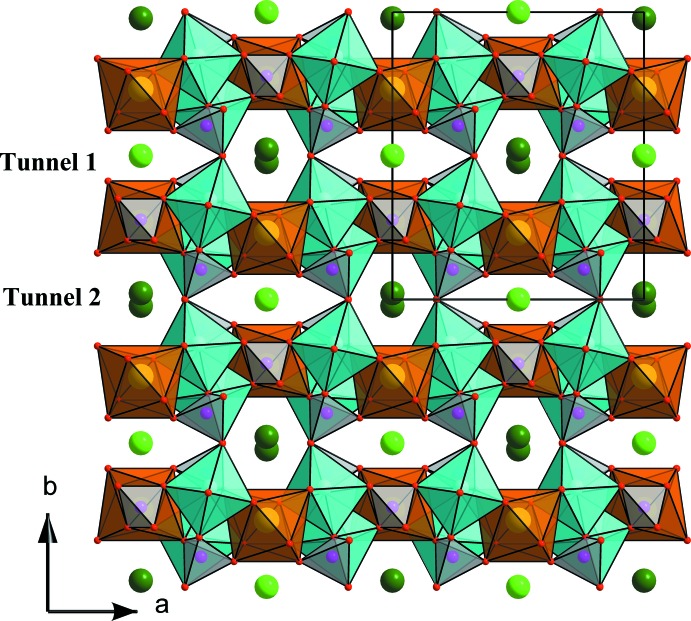
View of the alluaudite structure in the *ab* plane. The polyhedra represent a chain of *M*O_6_ octa­hedra parallel to [101]; Tunnel 1 (light-green atoms) and Tunnel 2 (dark-green atoms).

**Figure 4:**
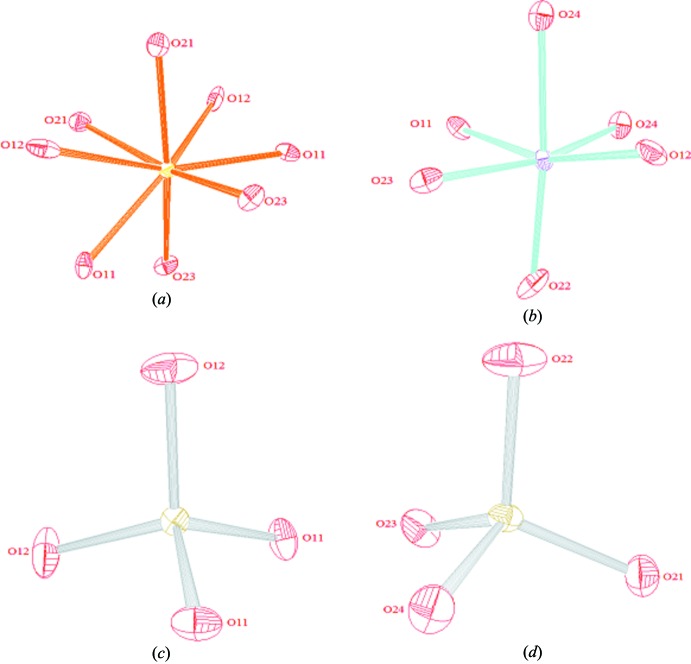
The environment of atoms (*a*) Ca, (*b*) Mn/Fe, (*c*) P1 and (*d*) P2.

The structure of the title compound consists of infinite chains (Fig. 1 ▸) formed by a succession of pairs of *M*(2)O_6_ octa­hedra linked together by common edges and sharing edges with a strongly distorted *M*(1)O_8_ polyhedron. Connected equivalent chains through the PO_4_ tetra­hedra lead to the formation of sheets stacked parallel to the *ac* plane (Fig. 2 ▸) and inter­connected along the *b* axis by PO_4_ tetra­hedra. The resulting three-dimensional anionic framework exhibits two kinds of tunnels parallel to the *c* axis situated at (1/2, 0, *z*) and (0, 0, *z*) (Fig. 3 ▸) and occupied by the Na^+^ ions. Fig. 4 ▸ shows the displacement ellipsoids of the coordination polyhedra of Ca, Mn/Fe, P1 and P2.

The *M*(2)—O distances and the O—*M*(2)—O angles range from 2.027 (2) to 2.246 (2) Å and from 80.11 (9) to 174.29 (9)°, respectively. This dispersion evidences an important distortion of the *M*(2)O_6_ octa­hedron due to edge-sharing. The *M*(1)O_8_ polyhedron is also very distorted as indicated by the *M*(1)—O distances and the O—*M*(1)—O angles which vary from 2.336 (2) to 2.951 (3) Å and from 54.00 (8) to 161.85 (8)°, respectively. In the P1O_4_ and P2O_4_ tetra­hedra, the P—O distances vary between 1.521 (2) and 1.547 (2) Å. Their mean distances 〈P1—O〉= 1.538 (2) Å and 〈P2—O〉= 1.537 (2) Å are in a good accordance with the value of 1.537 Å calculated by Baur (1974 ▸) for monophosphate groups.

**Figure 5:**
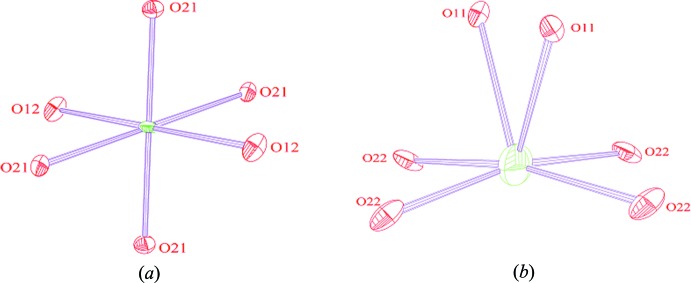
The environment of cations (*a*) Na1 and (*b*) Na2.

Assuming sodium–oxygen distances below 3.0, both the Na1 and Na2 sites are surrounded by six oxygen atoms. Their environments approximate strongly distorted octa­hedra (Fig. 5 ▸). Note that in the ideal alluaudite-type structure, both *X*(2) and *X*(1) sites are eightfold coordinated, such as for example in Na_2_Mn_2_Fe(PO_4_)_3_ and Na_2_Cd_2_Fe(PO_4_)_3_ (Hidouri *et al.*, 2011 ▸). However, in Na_4_CaFe_4_(PO_4_)_6_ (Hidouri *et al.*, 2004 ▸), the coordination numbers of the *X*(1) and *X*(2) sites are eight and six, respectively. The decrease of the *X*(2) coordination number seems to be related to the presence of calcium (0.5 Ca + 0.5 Na) in the *M*(1) site. In the title compound, the decrease of the coordination numbers from eight to six for both the *X*(1) and *X*(2) sites is probably related to the increase of the calcium content in the *M*(1) site, which becomes exclusively occupied by calcium.

## Synthesis and crystallization   

Single crystals of the title compound were obtained in a flux of sodium dimolybdate Na_2_Mo_2_O_7_. A starting mixture of appropriate amounts of Fe(NO_3_)_3_·9H_2_O (3.999 g); Mn(NO_3_)_2_·6H_2_O (2.472 g); CaCO_3_ (0.985 g); (NH_4_)_2_HPO_4_ (3.921 g); Na_2_CO_3_ (1.845 g) and MoO_3_ (2.148 g) was dissolved in nitric acid and then dried for 24 h at 353 K. The dry residue was well ground in an agate mortar and was gradually heated up to 873 K in a platinum crucible to evacuate the decomposition products NH_3_, CO_2_ and H_2_O. Then, the obtained product was melted for 1 h at 1073 K and was cooled slowly to 473 K at a rate of 10 K h^−1^. Finally, hexa­gonally shaped brown crystals of Na_2_CaMnFe(PO_4_)_3_ were obtained after washing the mixture with boiling water.

## Refinement   

Crystal data, data collection and structure refinement details are summarized in Table 1 ▸. The refinement was performed on the basis of electrical neutrality and previous work. Application of direct methods revealed the position of the site, labeled *M*(2), statistically occupied by the Fe^3+^ and Mn^2+^ ions. This distribution was supported by the *M*(2)—O distances, which range between those of Mn—O and Fe—O observed in similar environments.

## Supplementary Material

Crystal structure: contains datablock(s) global, I. DOI: 10.1107/S2056989016017771/br2263sup1.cif

Structure factors: contains datablock(s) I. DOI: 10.1107/S2056989016017771/br2263Isup2.hkl

CCDC reference: 1515407

Additional supporting information: crystallographic information; 3D view; checkCIF report

## Figures and Tables

**Table 1:** Experimental details

Crystal data	
Chemical formula	Na_2_CaMnFe(PO_4_)_3_
*M*_r_	481.76
Crystal system, space group	Monoclinic, *C*2/*c*
Temperature (K)	293
*a*, *b*, *c* (Å)	12.283 (1), 12.736 (1), 6.494 (5)
β (°)	114.76 (3)
*V* (Å^3^)	922.5 (7)
*Z*	4
Radiation type	Mo *K*α
μ (mm^−1^)	4.19
Crystal size (mm)	0.22 × 0.14 × 0.07

Data collection	
Diffractometer	Enraf–Nonius TurboCAD-4
Absorption correction	ψ scan (North *et al.*, 1968 ▸)
*T*_min_, *T*_max_	0.514, 0.689
No. of measured, independent and observed [*I* > 2σ(*I*)] reflections	1780, 1333, 1139
*R*_int_	0.023
(sin θ/λ)_max_ (Å^−1^)	0.702

Refinement	
*R*[*F* ^2^ > 2σ(*F* ^2^)], *wR*(*F* ^2^), *S*	0.028, 0.081, 1.07
No. of reflections	1333
No. of parameters	97
No. of restraints	2
Δρ_max_, Δρ_min_ (e Å^−3^)	0.63, −0.90

Computer programs: *CAD-4 EXPRESS* (Enraf–Nonius, 1994 ▸), *XCAD4* (Harms & Wocadlo, 1995 ▸), *SIR92* (Altomare *et al.*, 1993 ▸), *SHELXL2014* (Sheldrick, 2015 ▸), *DIAMOND* (Brandenburg, 1999 ▸) and *WinGX* (Farrugia, 2012 ▸).

## supplementary crystallographic information

### Crystal data

**Table:**

Na_2_CaMnFe(PO_4_)_3_	*F*(000) = 936
*M**_r_* = 481.76	*D*_x_ = 3.469 Mg m^−^^3^
Monoclinic, *C*2/*c*	Mo *K*α radiation, λ = 0.71073 Å
*a* = 12.283 (1) Å	Cell parameters from 25 reflections
*b* = 12.736 (1) Å	θ = 8.0–14.7°
*c* = 6.494 (5) Å	µ = 4.19 mm^−^^1^
β = 114.76 (3)°	*T* = 293 K
*V* = 922.5 (7) Å^3^	Prism, brown
*Z* = 4	0.22 × 0.14 × 0.07 mm

### Data collection

**Table:**

Enraf–Nonius TurboCAD-4 diffractometer	*R*_int_ = 0.023
Radiation source: fine-focus sealed tube	θ_max_ = 30.0°, θ_min_ = 2.4°
non–profiled ω/2τ scans	*h* = −17→16
Absorption correction: ψ scan (North *et al.*, 1968)	*k* = −1→17
*T*_min_ = 0.514, *T*_max_ = 0.689	*l* = −1→9
1780 measured reflections	2 standard reflections every 60 min
1333 independent reflections	intensity decay: none
1139 reflections with *I* > 2σ(*I*)	

### Refinement

**Table:**

Refinement on *F*^2^	2 restraints
Least-squares matrix: full	*w* = 1/[σ^2^(*F*_o_^2^) + (0.0351*P*)^2^ + 3.0677*P*] where *P* = (*F*_o_^2^ + 2*F*_c_^2^)/3
*R*[*F*^2^ > 2σ(*F*^2^)] = 0.028	(Δ/σ)_max_ < 0.001
*wR*(*F*^2^) = 0.081	Δρ_max_ = 0.63 e Å^−^^3^
*S* = 1.07	Δρ_min_ = −0.90 e Å^−^^3^
1333 reflections	Extinction correction: SHELXL2014 (Sheldrick, 2015), Fc^*^=kFc[1+0.001xFc^2^λ^3^/sin(2θ)]^-1/4^
97 parameters	Extinction coefficient: 0.0026 (4)

### Special details

**Table:**

Geometry. All esds (except the esd in the dihedral angle between two l.s. planes) are estimated using the full covariance matrix. The cell esds are taken into account individually in the estimation of esds in distances, angles and torsion angles; correlations between esds in cell parameters are only used when they are defined by crystal symmetry. An approximate (isotropic) treatment of cell esds is used for estimating esds involving l.s. planes.

### Fractional atomic coordinates and isotropic or equivalent isotropic displacement parameters (Å^2^)

**Table:**

	*x*	*y*	*z*	*U*_iso_*/*U*_eq_	Occ. (<1)
Na1	0.5000	0.0000	0.0000	0.0223 (4)	
Na2	0.0000	0.0217 (2)	0.7500	0.0464 (7)	
Ca	0.0000	0.26845 (6)	0.2500	0.01178 (18)	
Mn	0.22734 (3)	0.15466 (3)	0.14341 (7)	0.01013 (14)	0.4999 (3)
Fe	0.22734 (3)	0.15466 (3)	0.14341 (7)	0.01013 (14)	0.5001 (2)
P1	0.0000	0.27735 (8)	0.7500	0.0081 (2)	
O11	0.05225 (18)	0.20662 (17)	0.9616 (3)	0.0139 (4)	
O12	0.0910 (2)	0.35033 (18)	0.7174 (4)	0.0204 (5)	
P2	0.23941 (6)	−0.10428 (6)	0.13282 (11)	0.00951 (17)	
O21	0.37036 (19)	−0.08841 (17)	0.1794 (4)	0.0160 (4)	
O22	0.1756 (2)	0.00101 (19)	0.1190 (4)	0.0228 (5)	
O23	0.1718 (2)	−0.16191 (17)	−0.0952 (4)	0.0165 (4)	
O24	0.23187 (19)	−0.17266 (18)	0.3233 (4)	0.0164 (4)	

### Atomic displacement parameters (Å^2^)

**Table:**

	*U*^11^	*U*^22^	*U*^33^	*U*^12^	*U*^13^	*U*^23^
Na1	0.0296 (10)	0.0080 (8)	0.0110 (8)	−0.0041 (7)	−0.0095 (7)	0.0027 (6)
Na2	0.0339 (13)	0.0492 (16)	0.0406 (15)	0.000	0.0004 (11)	0.000
Ca	0.0109 (3)	0.0092 (4)	0.0175 (4)	0.000	0.0082 (3)	0.000
Mn	0.0079 (2)	0.0119 (2)	0.0101 (2)	−0.00091 (14)	0.00324 (16)	−0.00062 (14)
Fe	0.0079 (2)	0.0119 (2)	0.0101 (2)	−0.00091 (14)	0.00324 (16)	−0.00062 (14)
P1	0.0079 (4)	0.0090 (4)	0.0060 (4)	0.000	0.0016 (3)	0.000
O11	0.0132 (9)	0.0180 (10)	0.0082 (8)	−0.0031 (8)	0.0023 (7)	0.0036 (8)
O12	0.0163 (10)	0.0184 (11)	0.0255 (12)	−0.0014 (8)	0.0076 (9)	0.0111 (9)
P2	0.0116 (3)	0.0087 (3)	0.0062 (3)	0.0015 (2)	0.0017 (2)	0.0004 (2)
O21	0.0143 (9)	0.0160 (10)	0.0160 (10)	−0.0034 (8)	0.0049 (8)	−0.0015 (8)
O22	0.0291 (12)	0.0189 (11)	0.0173 (11)	0.0124 (9)	0.0064 (10)	−0.0018 (9)
O23	0.0211 (10)	0.0138 (10)	0.0094 (9)	−0.0034 (8)	0.0012 (8)	−0.0015 (8)
O24	0.0159 (9)	0.0212 (11)	0.0114 (10)	−0.0005 (9)	0.0052 (8)	0.0028 (8)

### Geometric parameters (Å, º)

**Table:**

Na1—O21^i^	2.315 (2)	Ca—O11^x^	2.355 (3)
Na1—O21^ii^	2.315 (2)	Ca—O12^vi^	2.951 (3)
Na1—O12^iii^	2.357 (2)	Ca—O12	2.951 (3)
Na1—O12^iv^	2.357 (2)	Mn—O12^xv^	2.027 (2)
Na1—O21^v^	2.591 (2)	Mn—O22	2.043 (3)
Na1—O21	2.591 (2)	Mn—O23^ix^	2.080 (3)
Na2—O22^vi^	2.477 (3)	Mn—O11^xiv^	2.081 (2)
Na2—O22^vii^	2.477 (3)	Mn—O24^i^	2.115 (3)
Na2—O22^viii^	2.645 (3)	Mn—O24^xi^	2.246 (2)
Na2—O22^ix^	2.645 (3)	P1—O12^x^	1.535 (2)
Na2—O11^x^	2.667 (3)	P1—O12	1.535 (2)
Na2—O11	2.667 (3)	P1—O11	1.541 (2)
Ca—O21^xi^	2.336 (2)	P1—O11^x^	1.541 (2)
Ca—O21^xii^	2.336 (2)	P2—O21	1.521 (2)
Ca—O23^xiii^	2.351 (2)	P2—O22	1.537 (2)
Ca—O23^ix^	2.351 (2)	P2—O23	1.546 (2)
Ca—O11^xiv^	2.355 (3)	P2—O24	1.547 (2)
			
O21^i^—Na1—O21^ii^	180.00 (13)	O23^ix^—Ca—O11^x^	87.80 (8)
O21^i^—Na1—O12^iii^	96.91 (9)	O11^xiv^—Ca—O11^x^	140.93 (11)
O21^ii^—Na1—O12^iii^	83.09 (9)	O21^xi^—Ca—O12^vi^	81.93 (8)
O21^i^—Na1—O12^iv^	83.09 (9)	O21^xii^—Ca—O12^vi^	65.72 (7)
O21^ii^—Na1—O12^iv^	96.91 (9)	O23^xiii^—Ca—O12^vi^	83.03 (8)
O12^iii^—Na1—O12^iv^	180.00 (13)	O23^ix^—Ca—O12^vi^	121.96 (7)
O21^i^—Na1—O21^v^	72.85 (9)	O11^xiv^—Ca—O12^vi^	54.00 (8)
O21^ii^—Na1—O21^v^	107.15 (9)	O11^x^—Ca—O12^vi^	145.50 (7)
O12^iii^—Na1—O21^v^	72.01 (9)	O21^xi^—Ca—O12	65.72 (7)
O12^iv^—Na1—O21^v^	107.99 (9)	O21^xii^—Ca—O12	81.93 (8)
O21^i^—Na1—O21	107.15 (9)	O23^xiii^—Ca—O12	121.96 (7)
O21^ii^—Na1—O21	72.85 (9)	O23^ix^—Ca—O12	83.03 (8)
O12^iii^—Na1—O21	107.99 (9)	O11^xiv^—Ca—O12	145.50 (7)
O12^iv^—Na1—O21	72.01 (9)	O11^x^—Ca—O12	54.00 (8)
O21^v^—Na1—O21	180.0	O12^vi^—Ca—O12	138.61 (10)
O22^vi^—Na2—O22^vii^	167.80 (17)	O12^xv^—Mn—O22	104.67 (10)
O22^vi^—Na2—O22^viii^	78.62 (9)	O12^xv^—Mn—O23^ix^	108.27 (10)
O22^vii^—Na2—O22^viii^	100.03 (9)	O22—Mn—O23^ix^	84.73 (9)
O22^vi^—Na2—O22^ix^	100.03 (9)	O12^xv^—Mn—O11^xiv^	161.07 (9)
O22^vii^—Na2—O22^ix^	78.62 (9)	O22—Mn—O11^xiv^	92.66 (9)
O22^viii^—Na2—O22^ix^	167.47 (16)	O23^ix^—Mn—O11^xiv^	80.46 (9)
O22^vi^—Na2—O11^x^	70.80 (8)	O12^xv^—Mn—O24^i^	87.98 (10)
O22^vii^—Na2—O11^x^	121.10 (12)	O22—Mn—O24^i^	99.38 (10)
O22^viii^—Na2—O11^x^	102.10 (9)	O23^ix^—Mn—O24^i^	161.79 (9)
O22^ix^—Na2—O11^x^	89.04 (9)	O11^xiv^—Mn—O24^i^	81.62 (9)
O22^vi^—Na2—O11	121.10 (12)	O12^xv^—Mn—O24^xi^	80.11 (9)
O22^vii^—Na2—O11	70.80 (8)	O22—Mn—O24^xi^	174.29 (9)
O22^viii^—Na2—O11	89.04 (9)	O23^ix^—Mn—O24^xi^	90.82 (8)
O22^ix^—Na2—O11	102.10 (9)	O11^xiv^—Mn—O24^xi^	83.06 (8)
O11^x^—Na2—O11	55.90 (11)	O24^i^—Mn—O24^xi^	83.79 (9)
O21^xi^—Ca—O21^xii^	77.41 (11)	O12^x^—P1—O12	105.48 (19)
O21^xi^—Ca—O23^xiii^	161.85 (8)	O12^x^—P1—O11	106.63 (13)
O21^xii^—Ca—O23^xiii^	87.17 (8)	O12—P1—O11	114.95 (12)
O21^xi^—Ca—O23^ix^	87.17 (8)	O12^x^—P1—O11^x^	114.95 (12)
O21^xii^—Ca—O23^ix^	161.85 (8)	O12—P1—O11^x^	106.63 (13)
O23^xiii^—Ca—O23^ix^	109.49 (11)	O11—P1—O11^x^	108.44 (18)
O21^xi^—Ca—O11^xiv^	91.53 (8)	O21—P2—O22	111.53 (14)
O21^xii^—Ca—O11^xiv^	119.68 (8)	O21—P2—O23	110.66 (13)
O23^xiii^—Ca—O11^xiv^	87.80 (8)	O22—P2—O23	107.57 (13)
O23^ix^—Ca—O11^xiv^	69.66 (8)	O21—P2—O24	109.17 (13)
O21^xi^—Ca—O11^x^	119.68 (8)	O22—P2—O24	109.76 (14)
O21^xii^—Ca—O11^x^	91.53 (8)	O23—P2—O24	108.08 (13)
O23^xiii^—Ca—O11^x^	69.66 (8)		

Symmetry codes: (i) *x*, −*y*, *z*−1/2; (ii) −*x*+1, *y*, −*z*+1/2; (iii) *x*+1/2, −*y*+1/2, *z*−1/2; (iv) −*x*+1/2, *y*−1/2, −*z*+1/2; (v) −*x*+1, −*y*, −*z*; (vi) −*x*, *y*, −*z*+1/2; (vii) *x*, *y*, *z*+1; (viii) −*x*, −*y*, −*z*+1; (ix) *x*, −*y*, *z*+1/2; (x) −*x*, *y*, −*z*+3/2; (xi) −*x*+1/2, *y*+1/2, −*z*+1/2; (xii) *x*−1/2, *y*+1/2, *z*; (xiii) −*x*, −*y*, −*z*; (xiv) *x*, *y*, *z*−1; (xv) −*x*+1/2, −*y*+1/2, −*z*+1.
